# The Sequential Roles of Psychological Flexibility and Positivity in the Association Between Post-Traumatic Stress Symptoms and Depressive Symptoms Among Earthquake Survivors in Türkiye

**DOI:** 10.3390/ejihpe16100158

**Published:** 2026-10-07

**Authors:** Adem Peker, Serkan Cengiz, Yüksel Eroğlu, Carlos Laranjeira, Murat Yıldırım

**Affiliations:** 1 Department of Counseling and Guidance, Faculty of Education, Ataturk University, 25240 Erzurum, Türkiye; 2 Department of Psychology, Faculty of Science and Letters, Agri Ibrahim Cecen University, Fırat Mahallesi Yeni Üniversite Caddesi No: 2 AE/1, 04100 Merkez, Agri, Türkiye; 3 Department of Counseling and Guidance, Faculty of Education, Afyon Kocatepe University, 03200 Afyon, Türkiye; 4 University of Leiria and Oeste, Rua General Norton de Matos, 2411-901 Leiria, Portugal; 5 Centre for Innovative Care and Health Technology (ciTechCare), Campus 5, Rua das Olhalvas, 2414-016 Leiria, Portugal; 6 Comprehensive Health Research Centre (CHRC), University of Évora, 7000-801 Évora, Portugal; 7 Department of Psychology, Faculty of Arts and Sciences, Haliç University, 34060 Istanbul, Türkiye; 8 Psychology Research Center, Khazar University, AZ1096 Baku, Azerbaijan

**Keywords:** PTSD, psychological flexibility, positivity, depression, earthquake

## Abstract

Earthquakes can have substantial consequences for psychological well-being, including post-traumatic stress and depressive symptoms. Drawing on Acceptance and Commitment Therapy (ACT), which conceptualizes psychological flexibility as a central process in adaptive psychological functioning, this study examined the sequential mediating roles of psychological flexibility and positivity in the association between post-traumatic stress symptoms and depressive symptoms among survivors of the two major earthquakes that struck Türkiye on 6 February 2023. This cross-sectional study included 834 earthquake survivors (*M*age = 26.7; 71.1% female) who were assessed approximately three months after the earthquakes. Participants completed validated self-report measures of post-traumatic stress symptoms, psychological flexibility, positivity, and depressive symptoms. Depressive symptoms were assessed using the 12-item depression subscale of the Brief Symptom Inventory and were analyzed as a continuous variable. Correlation and sequential mediation analyses were conducted, with indirect effects evaluated using bootstrapping. Post-traumatic stress symptoms were positively associated with depressive symptoms and negatively associated with psychological flexibility and positivity. Psychological flexibility was positively associated with positivity and negatively associated with depressive symptoms, while positivity was negatively associated with depressive symptoms. Sequential mediation analysis indicated that psychological flexibility and positivity mediated the association between post-traumatic stress symptoms and depressive symptoms. These findings suggest that psychological flexibility and positivity may represent relevant psychological processes in the association between post-traumatic stress and depressive symptoms among earthquake survivors and may inform post-disaster psychological support.

## 1. Introduction

On 6 February 2023, two major earthquakes struck southeastern Türkiye, causing widespread loss of life, displacement, and disruption across the affected region. Beyond their immediate physical and socioeconomic consequences, major earthquakes can be followed by substantial psychological distress among survivors. Previous research has documented post-traumatic stress and depressive symptoms as two of the most examined psychological outcomes following earthquake exposure (Gao et al., 2019; Ma et al., 2022; Tang et al., 2018). These symptom domains are particularly important because they frequently co-occur and may contribute to persistent difficulties in psychological adjustment after traumatic events. Understanding the psychological processes associated with their co-occurrence may therefore provide a more comprehensive account of post-disaster mental health than examining either symptom domain in isolation.

Acceptance and Commitment Therapy (ACT) conceptualizes psychological flexibility as a central process underlying adaptive psychological functioning (S. C. Hayes et al., 2006). Psychological flexibility involves the capacity to remain in contact with present-moment experiences, including difficult thoughts, emotions, memories, and bodily sensations, while acting in ways that are consistent with personally meaningful values. Conversely, psychological inflexibility involves rigid attempts to avoid, suppress, or control unwanted internal experiences and is considered an important process underlying psychological distress within the ACT framework (S. C. Hayes et al., 2006). Following a traumatic event such as an earthquake, survivors may attempt to avoid or control distressing internal experiences related to loss and trauma. From an ACT perspective, persistent experiential avoidance and psychological inflexibility may be associated with greater psychological distress, including depressive symptoms. Accordingly, psychological flexibility may be particularly relevant when examining psychological adjustment following traumatic experiences.

### 1.1. PTSD and Depression

Post-traumatic stress disorder (PTSD) is a trauma- and stressor-related disorder that may develop following exposure to actual or threatened death, serious injury, or sexual violence. According to the DSM-5-TR, PTSD symptoms are organized into four major clusters: intrusion symptoms, avoidance, negative alterations in cognitions and mood, and alterations in arousal and reactivity (American Psychiatric Association, 2022). Importantly, the negative alterations in cognitions and mood cluster (Criterion D) includes symptoms such as persistent negative emotional states, diminished interest or participation in significant activities, feelings of detachment or estrangement from others, and difficulty experiencing positive emotions. Thus, some features of PTSD may conceptually and phenomenologically overlap with depressive symptoms. Nevertheless, PTSD and depressive disorders remain distinct diagnostic constructs, and PTSD diagnosis requires consideration of the complete diagnostic criteria rather than the presence of depressive or negative-affect symptoms alone.

This distinction is particularly relevant in post-disaster research. Previous research on earthquake survivors has identified several factors associated with post-traumatic stress symptoms, including greater trauma exposure, loss of family members, communication difficulties, and reduced social support (Cerdá et al., 2013; Stimpson, 2006). Previous research has reported considerable variability in post-earthquake PTSD symptoms, ranging from 15% to 48% in adults (Goenjian et al., 2018), while studies following the Nepal earthquakes have reported rates ranging from 5% to 43% (Kane et al., 2018; Sharma & Kar, 2019). Such estimates should be interpreted in light of differences in assessment methods, diagnostic criteria, timing of assessment, and whether PTSD was established through clinical interviews or symptom-based screening instruments.

PTSD and depression frequently co-occur following traumatic experiences, and their association may partly reflect shared features of negative affect and mood disturbance. This overlap is particularly relevant to DSM-5 Criterion D, which encompasses negative alterations in cognition and mood. At the same time, empirical evidence indicates that post-traumatic stress and depressive symptoms represent distinguishable, although strongly related, psychological outcomes following trauma (Gonzalez et al., 2016; Olff et al., 2014). Ahmadi et al. (2024) found that stress experienced following traumatic exposure was associated with depressive symptoms, and a growing body of research indicates that depression is common among trauma-exposed individuals (Cao et al., 2015; Rytwinski et al., 2013).

Avoidance is a core symptom cluster of PTSD within the DSM-5-TR framework (American Psychiatric Association, 2022). ACT does not conceptualize avoidance as unique to PTSD; rather, it provides a broader functional account of experiential avoidance as attempts to escape, suppress, or control unwanted internal experiences (Orsillo & Batten, 2005). Following an earthquake, survivors may attempt to avoid distressing thoughts, emotions, memories, or physical sensations associated with the traumatic event and its aftermath. When such avoidance becomes rigid and persistent, it may be associated with greater post-traumatic stress and depressive symptoms (Bardeen et al., 2013; Shenk et al., 2012). Thus, in the present study, the ACT framework is used not to define avoidance as a unique feature of PTSD, but to understand how psychological flexibility and experiential avoidance may be relevant to the association between post-traumatic stress and depressive symptoms.

### 1.2. Mediation of Psychological Flexibility and Positivity

Within ACT, psychological flexibility refers to the capacity to remain in contact with present-moment experiences while adapting behavior in ways that are consistent with personally meaningful values (S. C. Hayes et al., 1999). In the aftermath of an earthquake, survivors may experience distressing thoughts, emotions, and memories that are difficult to tolerate. Psychological flexibility may enable individuals to respond to these internal experiences with greater openness and acceptance rather than rigid attempts to avoid or control them. In turn, greater psychological flexibility may facilitate more adaptive emotional responses and positive appraisals. Consistent with this perspective, psychological flexibility has been associated with positive emotions and appraisals (Kashdan et al., 2020; Xia et al., 2020).

Caprara et al. (2017) stated that positivity is a concept that reflects a positive approach or emotional state. Positivity reflects a general tendency to view oneself, life, and the future favorably and has been associated with adaptive functioning in the face of adversity (Caprara et al., 2009). Additionally, one of the important psychological processes that ACT interventions focus on is the experience of “being present” (S. C. Hayes et al., 2006). This concept involves people’s efforts to consciously adapt to ongoing internal and external experiences. Previous studies have demonstrated potential positive effects of positivity on various domains of functioning, including physical health, occupational performance, social adjustment, and psychological well-being (Alessandri et al., 2012; Fagnani et al., 2014). On the other hand, decreased positivity may be effective in predicting depression (Collins et al., 2021; Wood & Joseph, 2010). Disaster-related experiences, including personal injury, loss, and displacement, may challenge individuals’ positive evaluations of themselves, their lives, and the future. In this context, positivity may function as a psychological resource associated with lower levels of depressive symptoms (Collins et al., 2021; Wood & Joseph, 2010). Accordingly, greater psychological flexibility may facilitate more adaptive engagement with distressing post-traumatic experiences, which may in turn be associated with greater positivity and fewer depressive symptoms. This theoretical sequence provides the rationale for examining psychological flexibility and positivity as sequential mediators of the association between post-traumatic stress symptoms and depressive symptoms.

### 1.3. Present Study

Strong earthquakes often cause serious mental health consequences. A recent study showed that the prevalence of PTSD after the earthquake was 46% (Qi et al., 2020). Although post-traumatic stress and depressive symptoms have been widely examined following earthquakes, less is known about psychological processes that may help account for the association between these symptoms. Evidence concerning these processes among Turkish adult earthquake survivors also remains limited. In addition, different studies have found a mediating effect of psychological flexibility on the relationship between PTSD and depression (Huang et al., 2022; Richardson & Jost, 2019). However, the mediating role of positivity on PTSD and depression has been less explored. Post-traumatic stress symptoms may be associated with lower psychological flexibility, which may limit individuals’ ability to respond adaptively to distressing internal experiences. Greater psychological flexibility, in turn, may be associated with more positive evaluations of the self, life, and future, whereas greater positivity may be associated with fewer depressive symptoms. Accordingly, the present study examines psychological flexibility and positivity as sequential mediators of the association between post-traumatic stress symptoms and depressive symptoms. 

All participants in the present study were survivors of the 2023 earthquakes; therefore, earthquake exposure itself was not modeled as a predictor. Rather, the study focused on individual differences in post-traumatic stress symptoms among earthquake survivors and examined whether their association with depressive symptoms was sequentially mediated by psychological flexibility and positivity. Accordingly, the proposed model was specified as post-traumatic stress symptoms → psychological flexibility → positivity → depressive symptoms. Accordingly, the present study aimed to examine the sequential mediating roles of psychological flexibility and positivity in the association between post-traumatic stress symptoms and depressive symptoms among survivors of the 2023 earthquakes in Türkiye. Considering previous research and theoretical concepts, this study put forward the following hypotheses:

**H1.**  *Post-traumatic stress symptoms are positively associated with depressive symptoms.*

**H2.**  *Psychological flexibility and positivity sequentially mediate the relationship between post-traumatic stress symptoms and depressive symptoms.*

## 2. Materials and Methods

This research adhered to the STROBE guidelines for cross-sectional studies.

### 2.1. Participants

The sample comprised 834 survivors of the 6 February 2023 earthquakes in Türkiye, including 593 women (71.1%) and 241 men (28.9%). Participants ranged in age from 18 to 70 years (*M*age = 26.7, *SD* = 9.1). Regarding educational attainment, 33 participants (4.0%) completed primary school, 31 (3.7%) secondary school, 129 (15.5%) high school, and 641 (76.9%) university education. Additionally, 50.7% of the participants scored at or above the TSSC screening cut-off score of 25, indicating probable PTSD rather than a clinically established diagnosis. Information about the participants is included in Table 1.

### 2.2. Measures

(a) Traumatic Stress Symptom Checklist (TSSC)

The TSSC is a validated self-report screening instrument designed to assess trauma-related symptoms among earthquake survivors (Başoğlu et al., 2001). The instrument consists of 23 items comprising a 17-item PTSD symptom subscale and a six-item depression subscale. Items are rated on a 4-point scale (0 = not bothered at all, 3 = very bothered). In the present study, only the 17-item PTSD symptom subscale was used; the six-item depression subscale was not administered. The PTSD symptom subscale yields a total score ranging from 0 to 51, with higher scores indicating greater post-traumatic stress symptom severity. Başoğlu et al. (2001) reported a cut-off score of 25 for identifying individuals with probable PTSD. Because the TSSC is a self-report screening instrument, scores at or above this cut-off were interpreted as indicating probable PTSD rather than a clinically established diagnosis. An example item is “I feel uncomfortable when anything reminds me of what I experienced with the earthquake.” In the present study, Cronbach’s alpha for the PTSD symptom subscale was 0.94.

(b) Acceptance and Action Questionnaire-II (AAQ-II)

This scale was adapted from Bond et al. (2011) to measure the psychological flexibility level of adult individuals. Adaptation of the scale to Turkish was carried out by Yavuz et al. (2016). The scale has a total of seven items clustered on a single dimension. Scale items are rated on a 7-point scale (1 = Never true, 7 = Always true). The total score ranges from 7 to 49. In its conventional scoring, higher AAQ-II scores reflect greater psychological inflexibility. For the present study, all items were reverse-scored so that higher total scores represented greater psychological flexibility. Thus, the direction of the correlations and regression coefficients reported in the present study reflects this reverse-scored psychological flexibility metric. In the present study, Cronbach’s alpha was 0.92.

(c) Positivity Scale (PS)

The PS was developed by Caprara et al. (2012). Adaptation work to the Turkish context was carried out by Çıkrıkçı et al. (2015). The scale consists of a total of eight items and has a 5-point Likert-type scoring structure (ranging from 1: Not at all appropriate to 5: Completely appropriate). The total score ranges from 8 to 40. High scores on the scale indicate high positivity. For this study, Cronbach’s alpha was 0.81.

(d) Brief Symptom Inventory (BSI)

The BSI, developed by Derogatis (1982) and adapted into Turkish by Şahin and Durak (1994), consists of 53 items across five subscales: anxiety, depression, negative self, somatization, and hostility. In the present study, only the 12-item depression subscale was used to assess depressive symptoms. Participants evaluated the items on a 5-point Likert scale from 0: Not at all to 4: Very much. The total score ranges from 0 to 48, with higher scores indicating greater depressive symptom severity. The depression subscale was used for the study (e.g., “feeling sad and sad”). High scores from the depression subscale indicate that the individual has a high level of depression. In the present study, Cronbach’s alpha for the 12-item depression subscale was 0.93. In this study, we used the arithmetic mean and standard deviation scores to determine the depression level of the participants. In the present study, depressive symptoms were analyzed as a continuous variable using the total score of the 12-item BSI depression subscale, with higher scores indicating greater depressive symptom severity.

### 2.3. Procedures

Ethical approval for this study was granted by the Atatürk University Educational Sciences Unit Ethics Board (Meeting No. 04, Decision No. 13, dated 30 March 2023). Study data were collected approximately three months after the earthquake, between May 2023 and June 2023. After the earthquake, many students continued their education at different universities. These students were also at the universities where the researchers worked. The researchers administered the surveys to the affected students and then asked them to share them with their friends who were also affected by the earthquake using the snowball sampling method. These students also voluntarily delivered the surveys they received online to their families and friends who directly experienced the earthquake. Informed consent was obtained from all participants. The following criteria were applied for inclusion in the study: (i) participants had to be 18 years of age or older; (ii) they had to be from 11 earthquake-affected cities; (iii) they had to not have a psychiatric diagnosis. The purpose of the study was written on the form, and voluntary participation consent was added. Participants were free to withdraw at any time. Surveys were coded to anonymize responses for subsequent analysis.

### 2.4. Data Analysis

Firstly, the differences in PTSD and depression levels of individuals who experienced an earthquake according to gender were examined with an independent-samples *t*-test. Then, differences in post-traumatic stress and depressive symptom scores across age groups were examined using analysis of variance (ANOVA). Pearson’s correlation analysis was used to examine relationships between the research variables. We used A. F. Hayes’ (2017) PROCESS 3.3 macro (Model 6) to detect the sequential mediation effect of psychological flexibility and positivity. First, the direct effect of PTSD levels of individuals who experienced an earthquake on depression was examined. Then, the sequential mediating effect of psychological flexibility and positivity between PTSD and depression was examined. A bootstrap sample of 5000 and a 95% confidence interval for estimated indirect effects were considered. To consider the results significant in the analysis, the lower and upper limits of the confidence intervals must not contain zero (Preacher & Hayes, 2008). We performed statistical analyses using the SPSS v24 program.

## 3. Results

The mean post-traumatic stress symptom score was 42.00 (*SD* = 13.19). Of the 834 participants, 423 (50.7%) scored at or above the TSSC screening cut-off of 25, indicating probable PTSD; this classification should not be interpreted as a clinical diagnosis. The mean BSI depressive symptom score was 23.40 (*SD* = 12.59), and depressive symptoms were retained as a continuous variable in the analyses.

### 3.1. Findings Regarding the Differences in PTSD and Depression Scores by Gender

The change in adult individuals’ PTSD and depression levels according to gender after the earthquake was determined by an independent samples *t*-test. As a result of the analysis, it was revealed that women’s traumatic stress symptoms and depression levels were higher than men’s after the earthquake (t = 7.35, 7.67; *p* < 0.5). The results are in Table 2.

### 3.2. Findings Regarding the Differences in PTSD and Depression Scores According to Age

The significant difference between the PTSD and depression levels of adult individuals according to their age status after the earthquake was examined by analysis of variance. The results showed that there was a significant difference between the PTSD and depression scores of adult individuals according to their age levels. We performed the Tukey test to determine the ages at which this observed difference occurred. The analysis results reveal that individuals between the ages of 18–32 have significantly higher levels of PTSD and depression than those in other age groups (F = 14.59(2-831); *p* < 0.05; F = 37.76(2-831); *p* < 0.05). All results are in Table 3.

### 3.3. Preliminary Analyses

The skewness and kurtosis values of the variables in the study ranged from −0.984 to 0.061. These results show that the data meet the normality assumption (Tabachnick & Fidell, 2013). Additionally, the correlation results showed that PTSD was negatively associated with positivity (r = −0.34, *p* < 0.05) and psychological flexibility (r = −0.58, *p* < 0.05); It shows that they have a positive (r = 0.69, *p* < 0.05) relationship with depression. There are negative relationships between depression and positivity (r = −0.50, *p* < 0.05) and psychological flexibility (r = −0.76, *p* < 0.05). There is a positive relationship between positivity and flexibility (r = 0.48, *p* < 0.05). Preliminary analysis results are included in Table 4.

### 3.4. Mediation Analyses

**Figure 1:**
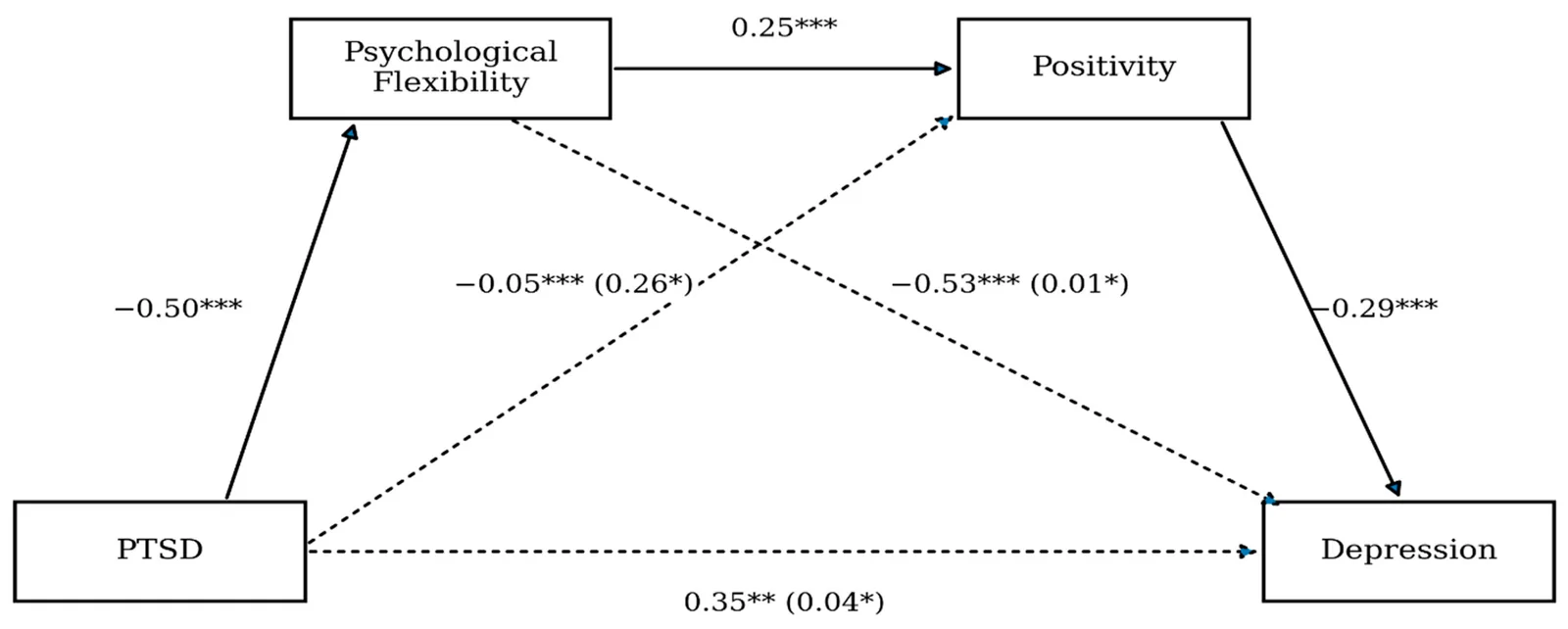
The proposed mediation model. *** *p* < 0.05. ** total effect, * indirect effect.

The results showed that post-traumatic stress symptoms significantly predicted depressive symptoms (β = 0.35, *p* < 0.05). Post-traumatic stress symptoms negatively predicted psychological flexibility (β = −0.50, *p* < 0.05) and positivity (β = −0.05, *p* < 0.05). Psychological flexibility positively predicted positivity (β = 0.25, *p* < 0.05) and negatively predicted depressive symptoms (β = −0.53, *p* < 0.05), whereas positivity negatively predicted depressive symptoms (β = −0.29, *p* < 0.05). When psychological flexibility and positivity were included in the model, the direct association between post-traumatic stress symptoms and depressive symptoms was reduced. The indirect-effect analysis indicated that psychological flexibility and positivity sequentially mediated this association (β = 0.036, 95% CI [0.0228, 0.0508]). The model accounted for 68.7% of the variance in depressive symptom scores. This mediation model explained 68.7% of the variance in depression among earthquake victims (See Figure 1). Standardized indirect values are reported in Table 5.

## 4. Discussion

### 4.1. Evaluation of PTSD and Depression in Terms of Demographic Characteristics

In the present study, 50.7% of earthquake survivors scored at or above the TSSC screening cut-off, indicating probable PTSD rather than a clinically established diagnosis. Additionally, the mean BSI depressive symptom score was 23.40 (*SD* = 12.59). Because depressive symptoms were analyzed as a continuous variable, participants were not classified into diagnostic or severity categories. Previous studies have reported considerable variation in post-traumatic stress and depressive symptoms following earthquakes. For example, Liu et al. (2018) reported PTSD in 23.4% of participants four years after an earthquake, whereas Marthoenis et al. (2019) reported post-traumatic stress symptoms in 58.3% and depressive symptoms in 16.8% of participants. Goodwin et al. (2020) reported a PTSD prevalence of 42.21% among Wenchuan earthquake survivors. Differences across studies may reflect variation in assessment instruments, time since the disaster, previous traumatic experiences, sample characteristics, and other sociodemographic factors.

In the present earthquake sample, women reported significantly higher post-traumatic stress and depressive symptom scores than men. This result is consistent with other studies showing that women experience more stress than men after earthquakes (Pan et al., 2015; Zhang et al., 2011). Liang (2017) reported that elderly females experienced more severe depression and anxiety than males. Jia et al. (2010) reported that older earthquake survivors suffered more from depression than younger adult survivors.

### 4.2. PTSD and Depression

Consistent with Hypothesis 1 and previous research, the present findings showed a positive association between post-traumatic stress symptoms and depressive symptoms in this earthquake sample. The strong association between post-traumatic stress and depressive symptoms should also be interpreted considering their partial symptom overlap. As noted above, DSM-5-TR Criterion D includes negative alterations in cognition and mood, some of which, such as diminished interest, persistent negative emotional states, and difficulty experiencing positive emotions, may overlap with depressive symptomatology (American Psychiatric Association, 2022). Therefore, the observed association may partly reflect shared affective features, while PTSD and depression remain conceptually distinct constructs. Importantly, the present findings are based on self-reported symptom severity and should not be interpreted as demonstrating an association between clinically established PTSD and a clinical diagnosis of depression. From an ACT perspective, experiential avoidance and psychological inflexibility may be relevant to the co-occurrence of post-traumatic stress and depressive symptoms. Greater experiential avoidance has been associated with psychological distress and depressive symptoms, particularly when individuals respond rigidly to difficult internal experiences (Zettle, 2015). Consistent with the present findings, previous studies have documented substantial associations between post-traumatic stress and depressive symptoms across trauma- and disaster-exposed populations (Janssen et al., 2015; Jin et al., 2018; Pan et al., 2015; Park & Bae, 2022). Pan et al. (2015) revealed that there were significant relationships between PTSD and depression after the Wenchuan earthquake. A previous study found positive relationships between PTSD and depression levels in disaster victims (Park & Bae, 2022). Previous research suggests that sociodemographic and disaster-related adversities, including socioeconomic difficulties and greater levels of personal and material loss, may be associated with greater psychological distress among earthquake survivors (Ali et al., 2012; Cénat & Derivois, 2014). Such factors may contribute to the broader context in which post-traumatic stress and depressive symptoms co-occur, but the present study does not establish that these adversities intensify post-traumatic stress symptoms.

### 4.3. Mediation of Psychological Flexibility and Positivity

The findings supported Hypothesis 2, indicating that psychological flexibility and positivity sequentially mediated the association between post-traumatic stress symptoms and depressive symptoms. These findings suggest that psychological flexibility and positivity may help account for this association. Koesten et al. (2009) state that flexibility will help increase well-being as it will contribute to the individual’s management of stress. Fu and Chow (2017) found that individuals with high psychological flexibility levels had more positive emotions than those with low psychological flexibility when injured during an earthquake.

Previous research has also linked optimism and positive psychological orientations with psychological adjustment following disaster exposure (Birkeland et al., 2017; Carbone & Echols, 2017; Gargano et al., 2019). Within the ACT framework, psychological flexibility may enable individuals to respond more openly and adaptively to difficult internal experiences rather than engaging in rigid avoidance (S. C. Hayes et al., 2006). Positivity may likewise represent a psychological resource associated with lower depressive symptom severity. Taken together, the present findings suggest that psychological flexibility and positivity may represent relevant sequential mediating processes in the association between post-traumatic stress symptoms and depressive symptoms.

From a theoretical perspective, the sequential mediation pattern extends the interpretation of post-disaster psychological adjustment beyond the direct association between post-traumatic stress and depressive symptoms. Within an ACT-informed framework, psychological flexibility reflects the capacity to respond more adaptively to distressing internal experiences rather than rigidly avoiding them (S. C. Hayes et al., 2006; Kashdan et al., 2020), whereas positivity reflects broader positive evaluations of the self, life, and future (Caprara et al., 2009, 2012). In this context, the present findings suggest that psychological flexibility and positivity may represent interconnected psychological processes in the association between post-traumatic stress and depressive symptoms among earthquake survivors. From an applied perspective, survivors experiencing these symptoms may benefit from psychological support that considers psychological flexibility and positivity. However, given the cross-sectional design, the observed sequential mediation should not be interpreted as evidence of causal relationships, and longitudinal and intervention studies are needed to determine whether changes in these processes preceded subsequent changes in symptoms.

The present study has several limitations. First, cross-sectional design does not permit conclusions about temporal or causal relationships among the study variables. Future longitudinal studies could examine changes in psychological symptoms and the proposed mediating processes over time. Second, post-traumatic stress and depressive symptoms were assessed using self-report instruments rather than structured clinician-administered diagnostic interviews. In particular, the TSSC is a screening instrument, and its cut-off indicates probable PTSD rather than a clinically confirmed diagnosis. Therefore, the proportion of participants scoring above the cut-off should not be interpreted as the prevalence of clinically diagnosed PTSD. In addition, the findings are based on the specific self-report measures used in the present study; alternative validated measures of post-traumatic stress symptoms, depressive symptoms, psychological flexibility, and positivity may yield somewhat different estimates of the observed associations. Third, the sample was relatively young (*M*age = 26.7 years), predominantly female (71.1%), and highly educated, with 76.9% of participants reporting a university degree. These characteristics may limit the generalizability of the findings to the broader population living in the earthquake-affected region. Future studies should therefore include more demographically diverse and representative samples. Fourth, pre-earthquake depressive symptoms were not assessed. Although participants reported no psychiatric diagnosis at the time of inclusion, this does not rule out the possibility of depressive symptoms before the earthquakes. Thus, the present findings cannot determine the extent to which depressive symptoms emerged following the earthquakes or reflected pre-existing vulnerability. Finally, the study did not include a comparable non-exposed control group. Consequently, the present findings cannot determine the extent to which the observed post-traumatic stress and depressive symptom levels were specifically attributable to earthquake exposure. Future research incorporating pre-disaster or longitudinal assessments and comparable non-exposed groups would provide stronger evidence regarding psychological changes associated with disaster exposure.

## 5. Conclusions

This study examined the sequential mediating roles of psychological flexibility and positivity in the association between post-traumatic stress symptoms and depressive symptoms among survivors of the 2023 earthquakes in Türkiye. The findings showed that post-traumatic stress symptoms were positively associated with depressive symptoms and that psychological flexibility and positivity sequentially mediated this association. These findings suggest that psychological flexibility and positivity may represent relevant psychological processes in the association between post-traumatic stress and depressive symptoms. Earthquake survivors experiencing these symptoms may therefore benefit from psychological interventions that support psychological flexibility and positivity. Given the cross-sectional design of the study, these findings should be interpreted as associations rather than evidence of causal or intervention effects.

## Figures and Tables

**Table 1:** Demographic Characteristics of the Respondents.

Variables	*N*	%
Gender		
Woman	593	71.1
Man	241	28.9
Age		
Mean ± *SD* of age	26.7 ± 9.1	
Graduation		
Primary school	33	4
Secondary school	31	3.7
High school	129	15.5
University	641	76.9

Note. *SD* = standard deviation.

**Table 2:** Gender Differences in PTSD and Depression Scores.

Variables	PTSD			Depression		
	Mean ± *SD*	t	*p*	Mean ± *SD*	t	*p*
Gender						
Woman	44.08 ± 12.69	7.35	0.01 *	25.46 ± 11.87	7.67	0.01 *
Man	36.89 ± 13.04			18.33 ± 12.91		

* *p* < 0.05.

**Table 3:** Associations Between Age Group and Mental Health Outcomes.

Variables	PTSD				Depression			
Age	Mean ± *SD*	F	I–J	*p*	Mean ± *SD*	F	I–J	*p*
18–32	43.37 ± 12.97	14.59	-	-	25.44 ± 12.12	37.76	-	-
33–47	37.68 ± 13.06		5.69	0.01 *	16.73 ± 12.08		8.71	0.01 *
48 and above	37.76 ± 12.75		5.60	0.04 *	18.15 ± 11.01		7.30	0.02 *

Note: PTSD = Posttraumatic stress disorder; * *p* < 0.05.

**Table 4:** Descriptive Statistics and Correlation Between the Variables.

Variables	1	2	3	4
1. PTSD	-			
2. Depression	0.69 *	-		
3. Positivity	−0.34 *	−0.50 *	-	
4. Psychological flexibility	−0.58 *	−0.76 *	0.48 *	-
Mean	42.00	23.40	24.85	31.28
Standard Deviation	13.19	12.59	6.65	11.29
Skewness	0.061	−0.103	−0.216	−0.276
Kurtosis	−0.984	−0.842	−0.312	−0.800
α	0.94	0.93	0.81	0.92

Note. PTSD = Posttraumatic stress disorder; * *p* < 0.05.

**Table 5:** Results showing direct, indirect and total effects.

Paths	Estimates	SE	CI (95%)	
Direct effects			LLCI	ULCI
PTSD→DEP	0.350	0.020	0.3041	0.3937
PTSD→PFL	−0.500	0.020	−0.5430	−0.4481
PTSD→PS	−0.050	0.020	−0.0822	−0.0086
PFL→PS	0.250	0.050	0.2092	0.2952
PS→DEP	−0.290	0.040	−0.3735	−0.2082
PFL→DEP	−0.530	0.030	−0.5823	−0.4699
Indirect effects				
PTSD→PFL→DEP	0.26	0.02	0.2189	0.3065
PTSD→PS→DEP	0.01	0.006	0.0016	0.0264
PTSD→PFL→PS→DEP	0.036	0.007	0.0228	0.0508
Total effect				
PTSD→DEP	0.31	0.02	0.2707	0.3529

Note: PTSD = Post-traumatic stress disorder, DEP = Depression, PFL = Psychological flexibility, PS = Positivity.

## Data Availability

The datasets generated during and/or analysed during the current study are available from the corresponding author upon reasonable request.

## Abbreviations

The following abbreviations are used in this manuscript:

ACT	Acceptance and Commitment Therapy
AAQ-II	Acceptance and Action Questionnaire-II
BSI	Brief Symptom Inventory
PTSD	Post-Traumatic Stress Disorder
PS	Positivity Scale
TSSC	Traumatic Stress Symptom Checklist
